# Differential neurocognitive profiles in adult attention-deficit/hyperactivity disorder subtypes revealed by the Cambridge Neuropsychological Test Automated Battery

**DOI:** 10.1007/s00406-023-01702-x

**Published:** 2023-11-18

**Authors:** Szilvia Somogyi, Tünde Kilencz, Katalin Szőcs, Izabella Klein, Lívia Balogh, Rebeka Molnár, Sára Bálint, Attila J. Pulay, Zsófia Nemoda, Máté Baradits, János M. Réthelyi

**Affiliations:** 1 Department of Psychiatry and Psychotherapy, Semmelweis University, Balassa utca 6, Budapest, 1083 Hungary; 2 Molecular Psychiatry and In Vitro Disease Modeling Research Group, Semmelweis University, Budapest, Hungary; 3 Department of Molecular Biology, Semmelweis University, Budapest, Hungary

**Keywords:** Adult ADHD, Neurocognitive impairment, CANTAB, Principal component analysis, Sustained attention, aADHD subtypes

## Abstract

Adult attention-deficit/hyperactivity disorder (aADHD) represents a heterogeneous entity incorporating different subgroups in terms of symptomatology, course, and neurocognition. Although neurocognitive dysfunction is generally associated with aADHD, its severity, association with self-reported symptoms, and differences between subtypes remain unclear. We investigated 61 outpatients (65.6% male, mean age 31.5 ± 9.5) diagnosed using DSM-5 criteria together with age-, sex-, and education-matched healthy controls (HC) (*n* = 58, 63.8% male, mean age 32.3 ± 9.6). Neurocognitive alterations were assessed using the Cambridge Neuropsychological Test Automated Battery (CANTAB) and compared between groups using the generalized linear model (GLM) method. Multivariate effects were tested by principal component analysis combined with multivariate pattern analysis. Self-reported symptom severity was tested for correlations with neurocognitive performance. GLM analyses revealed nominally significant differences between the aADHD and HC groups in several domains, however, only the Rapid Visual Information Processing measures survived correction, indicating impaired sustained attention and response inhibition in the aADHD group. Comparison of the predominantly inattentive and the hyperactive-impulsive/combined subtypes yielded nominally significant differences with higher levels of dysfunction in the inattentive group. In the stepwise discriminant analysis aADHD and HC groups were best separated with 2 factors representing sustained attention and reaction time. We found only weak correlations between symptom severity and CANTAB factors. aADHD patients are neuropsychologically heterogeneous and subtypes show different neurocognitive profiles. Differences between the aADHD and HC groups were driven primarily by the inattentive subtype. Sustained attention and its factor derivative showed the most significant alterations in aADHD patients.

**Supplementary Information:**

The online version contains supplementary material available at 10.1007/s00406-023-01702-x.

## Introduction

Attention deficit-hyperactivity disorder (ADHD) is a childhood-onset neurodevelopmental disorder. It is characterized by the core symptoms of inattention, impulsivity and motor hyperactivity [1], which can lead to decreased educational performance and social difficulties in children, and by persistence of symptoms, somatic and psychiatric comorbidity in adults [2–4]. The etiology of ADHD, although not fully understood, is considered multifactorial; common and rare genetic variants together with environmental effects are associated with increased disease risk, worse symptom profiles and severity in later life [5]. ADHD persists into adulthood in 35–50% of cases [6, 7], resulting in an adulthood prevalence around 2.5% worldwide [8, 9]. Adult ADHD (aADHD) is characterized by transitioned symptom presentation and functional consequences, including decreased higher education and work outcomes, interpersonal and family dysfunction. Moreover, in 30% and 18% of cases substance misuse [10] and criminal involvement [11] are also reported, respectively. Therefore, ADHD represents a major public health issue that affects a considerable patient population and their environments, both in children and adults. As a consequence, it needs to be targeted with complex clinical and psychosocial modalities and considered in the lifespan perspective [12–14]. Importantly, symptom severity, course and prognosis can be improved by efficient pharmacological and non-pharmacological interventions, given that the disorder is identified, diagnosed, and treated in proper time [15].

Neurocognitive impairment has been consistently described in aADHD in the domains of attention and sustained attention, reaction time variability, motor inhibition, and different subdomains of executive functioning, including cognitive flexibility, working memory, and delay discounting. However, cognitive heterogeneity has also been demonstrated by a systematic analysis, identifying a subgroup of aADHD patients without any significant, easily measurable cognitive dysfunction [16]. Alterations in response inhibition as the hypothesized main driver of deficiencies in ADHD [17], have been only supported partially by empirical data. This led to the refinement of the unifying neuropsychological models of ADHD, e.g., Sonuga-Barke described the dual-pathway model [18], where executive dysfunction and alterations of the reward circuit, i.e., delay aversion, together give rise to the complex symptomatology of ADHD. Indeed, executive dysfunction seems to be an overlapping phenotype in all age groups and phenotypic subgroups of ADHD, while delay aversion is a potential driver of impulsive behavior [19]. Another potential source of heterogeneity in adults compared to children, is more developed and individually varying compensation strategies. The clinical importance of neurocognitive impairment is underscored by longitudinal results, showing that persistence into and decreased functionality in adulthood is predicted by the severity of neuropsychological alterations [20, 21]. Recently, Onandia-Hinchado et al. reviewed all available studies investigating cognitive impairments in aADHD [22], showing the involvement of both attentional and multiple executive domains.

Decision making as a proxy for risk taking and impulsivity has also been investigated in aADHD, with a special emphasis on the association of delay discounting alterations and impulsivity as core symptoms. Impaired delay discounting has been proposed as an important feature of aADHD, but the results remain conflicting. Pollak et al. [23] demonstrated similar levels of risk taking with the Cambridge Gambling Test, indicative of equal sensitivity to risk in the aADHD and healthy control groups. In a recent review, the same group [24] showed that not risk taking per se, rather decision-making strategies are affected in aADHD, leading to real-life risk taking behaviors.

The Cambridge Neuropsychological Test Automated Battery (CANTAB) is used extensively for the assessment of neurocognitive performance in several mental disorders [25, 26]. The applied subtests of the CANTAB software are independent of culture and measure distinct neurocognitive functions including psychomotor speed, sustained attention, visual memory, executive functions, working memory and planning, semantic/verbal memory, decision making, response control, and social cognition. Application of the CANTAB software in aADHD demonstrated similar degree of heterogeneity of neurocognition within patients as previous studies. In a group of 474 DSM-IV diagnosed aADHD patients and 163 healthy controls Fried et al. [27] demonstrated that despite the differences of 7 CANTAB subtests in the fields of working memory and executive functioning, these results failed to discriminate effectively between patients and healthy controls. However, the CANTAB results were helpful in identifying the patient subgroup with executive functioning disorder, a finding with potential implications for the clinical management of aADHD patients. Therefore, we can conclude that the CANTAB software is not suitable for diagnosing aADHD, primarily because the diagnostic criteria are based on behavioral symptoms, and do not fully encompass cognitive alterations. However, the CANTAB software, due to the growing level of complexity in certain subtests, which necessitate adaptability and executive functions in subjects, can delineate subgroups within aADHD characterized by more profound cognitive dysfunction. This unique feature warrants interest for continued studies of aADHD with the same approach.

It remains unclear to what extent different cognitive domains exert independent effects, i.e., are the different alterations specific and non-overlapping in their consequences on outcomes and symptom severity. Interestingly, inconsistencies that have been shown in the above studies could also be explained by differential neurocognitive alterations in the subtypes of aADHD. The distinction between the predominantly inattentive (aADHD-I), predominantly hyperactive-impulsive (aADHD-HI), and combined presentation subtypes (aADHD-C) of aADHD, as defined by DSM-5 [1] is made according to symptom severity of these domains. In children and youth, Nikolas and Nigg [28] found that the neuropsychological performance of the ADHD-C group was worse than the ADHD-I group. However, earlier in a meta-analysis of 83 studies of childhood ADHD no differences were revealed in executive functions between the combined and inattentive subtypes [29]. In adults, Dobson-Patterson et al. [30] found that the aADHD-I group showed a clear separation in a multivariate comparison of tests assessing attention, memory and executive function, while the aADHD-C group did not separate from the healthy control group using the same approach. In contrast, Phillips et al. demonstrated worse performance in the domains of visual and verbal memory in aADHD-C individuals [3, 4, 31]. These findings are suggestive of the hypothesis that in aADHD inattentive patients are affected more severely, but only in certain domains of neurocognitive impairment. A complementary approach to group comparisons is to test for associations between neurocognitive alterations and clinical variables, such as self-reported symptom severity or comorbidities. In this regard there is a scarcity of data in the literature, however, the existing results have shown that subjective and objective measures of cognitive impairments do not show good correlation [32].

This study had three aims: First we sought to describe neurocognitive alterations and decision making in aADHD and its subtypes, compared to healthy controls, in a sample of Hungarian patients. Second, using a factor-analytic approach as a data-reduction technique we compared aADHD subtypes in terms of neurocognitive alterations after controlling for overlapping effects and using a stepwise multivariate pattern analysis. Therefore, non-specific, overlapping effects between cognitive tests were also considered. Third, we analyzed the correlations of self-reported symptom severity with neurocognition. The rationale behind this complex approach is to decrease the phenotypic heterogeneity characteristic of aADHD, by investigating differences in disease subgroups and associations with subjective symptom severity as a continuous variable. Thus, a better understanding of the associations between neurocognitive dysfunction and aADHD can be reached.

## Methods

### Sample recruitment and characteristics

Sixty-one aADHD patients (40 male, 21 female) and fifty-eight healthy control individuals (37 male, 21 female) matched for sex, age, and educational level were included in the study (Table 1). All examinations were carried out between January 2016 and June 2017. aADHD patients were recruited at the adult ADHD outpatient clinic of the Department of Psychiatry and Psychotherapy, Semmelweis University, Budapest, Hungary. The diagnosis of aADHD was originally established according to DSM-IV-R diagnostic criteria, later all data were reanalyzed using the DSM-5 as well, demonstrating that all patients fulfill the criteria of mid-severe aADHD. The diagnosis and aADHD subtypes were established by two experienced psychiatrists based on DSM-5 criteria using a detailed clinical interview and patient history. Screening for psychiatric comorbidities was carried out using the Hungarian versions [33, 34] of the MINI PLUS 5.0 [35, 36] and SCID-II [37] interviews. Exclusion criteria in the patient group were IQ under 70, a comorbid diagnosis of neurocognitive disorders, psychotic disorders, and severe neurological conditions. 59 of the ADHD patients were medicated, with 58 receiving methylphenidate; and 1 patient receiving bupropion. Patients taking stimulant treatment were off medication at least 24 h before testing.

**Table 1:** Clinical and demographic characteristics of the aADHD and healthy control (HC) groups

Characteristic	aADHD (*n* = 61)	HC (*n* = 58)	*F*/*χ*^2^/*t*	*p*
Age (years, SD)	31.49 (9.5)	32.33 (9.6)	0.475	0.635
Sex (M:F ratio)^a^	40:21	37:21	0.41	0.850
Level of education (E:S:H)^b^	4:28:29	0:24:34	2.891	0.089
Education (years, SD)	15.28 (2.6)	16.72 (2.5)	3.093	**0.002**
Subtypes (I:H:C)^c^	27:5:29			
IQ^d^				
Min	94	–		
Max	144	–		
Mean (SD)	125.76 (12.12)	–		
PQ − mean (SD)	124.07 (12.78)	–		
VQ − mean (SD)	124.31 (12.12)	–		
*Conners’ ADHD Rating Scale (CAARS)*				
Inattention/memory problems (CAARS-A)	26.77 (5.4)	11 (8.2)	10.102	**< 0.001**
Hyperactivity/restlessness (CAARS-B)	20.89 (6.8)	9.05 (4.73)	9.952	**< 0.001**
Impulsivity/emotional lability CAARS-C)	20.38 (7.7)	8.35 (5.9)	7.918	**< 0.001**
Problems with self-concept (CAARS-D)	11.18 (4.7)	4.62 (4.0)	7.236	**< 0.001**
DSM-IV inattentive symptoms (CAARS-E)	19.47 (4.9)	6.46 (5.7)	11,714	**< 0.001**
DSM-IV hyperactive-impulsive symptoms (CAARS-F)	15.28 (5.3)	5.26 (3.8)	9.675	**< 0.001**
DSM-IV ADHD symptoms total (CAARS-G)	39.59 (8.9)	13.44 (9.6)	13.344	**< 0.001**
ADHD index (CAARS-H)	24.31 (4.9)	8.18 (6.1)	13.167	**< 0.001**
*Symptom Check List (SCL-90-R)*				
Global Severity Index (GSI)	1.05 (0.6)	0.28 (0.29)	7.397	**< 0.001**
Somatisation	8.30 (6.6)	2.63 (3.7)	4.670	**< 0.001**
Obsessive compulsive	15.28 (7.6)	3.17 (3.1)	9.267	**< 0.001**
Interpersonal sensitivity	10.58 (8.2)	4.04 (4.17)	4.433	**< 0.001**
Depression	19.48 (11.9)	5.60 (7.4)	6.151	**< 0.001**
Anxiety	11.25 (6.9)	2.37 (2.6)	7.533	**< 0.001**
Hostility	5.50 (4.6)	1.51 (2.1)	4.889	**< 0.001**
Phobic anxiety	4.88 (2.8)	0.89 (1.5)	7.757	**< 0.001**
Paranoid ideation	5.50 (5.53)	1.49 (1.93)	4.302	**< 0.001**
Psychoticism	6.35 (7.0)	1.14 (2.3)	4.421	**< 0.001**
*Comorbidity*				
All	26/61	–		
Dyslexia/dysgraphia	4	–		
Depression	14	–		
Bipolar affective disorder	2	–		
Anxiety	9	–		
Personality disorder	2	–		
Substance use	3	–		
Other	2	–		
*Medication data*		–		
No medication	2	–		
Bupropion	1	–		
Methylphenidate	58	–		

^a^M = Male, F = Female

^b^For completed education, E = elementary, S = secondary, H = higher education

^c^aADHD subtypes, I = inattentive, H = hyperactive, C = combined

^d^(*n* = 54)

Healthy controls with negative psychiatric history were recruited from staff members, students and their acquaintances. They underwent a screening procedure and were excluded in case of positive neurological, psychiatric, or substance use disorder history, or a T-score above 70 on the Symptom Checklist 90-R (SCL-90-R) [38, 39]. Exclusion criteria in both groups were a positive history of severe head trauma and visual or movement impairment that could influence results during touch screen use. The study complied with the ethical standards of the Declaration of Helsinki, and received approval from the Hungarian Health Sciences Council Ethical Committee (IF-11390-8/2015 and IF-621-2/2017, modified by nrs. 25329-5/2018/EÜIG and 926-5/2018/EÜIG, respectively). All participants gave written informed consent.

### Examination procedure, clinical and neuropsychological variables

After written informed consent, participants in both groups were asked to complete two self-report questionnaires: the Conner’s Adult ADHD Rating Scale (CAARS) [40] and the Symptom Check List (SCL-90-R) [38, 39]. The Hungarian version of the CAARS 66 item self-report questionnaire was used to assess symptom severity [40–43]. The CAARS measures the severity of self-reported symptoms on seven subscales (CAARS-A: Inattention/Memory Problems; CAARS-B: Hyperactivity/Restlessness; CAARS-C: Impulsivity/Emotional Lability; CAARS-D: Problems with Self-Concept; CAARS-E: DSM-IV Inattentive Symptoms; CAARS-F: DSM-IV Hyperactive-Impulsive Symptoms, CAARS-G: DSM-IV Total ADHD Symptoms; CAARS-H: ADHD Index). All items represent a 4-point scale (values between 0 and 3). The Hungarian version of the SCL-90-R [38, 39, 44, 45] was used to assess general psychopathology. Healthy controls were excluded if they tested above the T-score of 70 in the Derogatis criteria. IQ scores were assessed only in patients using the WAIS-R [46].

### Cambridge Neuropsychological Test Automated Battery

All subjects underwent neurocognitive assessment using the Cambridge Neuropsychological Test Automated Battery (CANTAB EclipseTM 5.0, Cambridge Cognition, Cambridge, United Kingdom). CANTAB was originally developed to assess neurocognitive performance mainly of patients suffering from neurocognitive impairment [25, 26, 47]. Recently, it has been used to study several psychiatric disorders, and has been validated on several patient groups, resulting in good validity and reliability data. As of now, CANTAB has been used and proved to be a useful tool to assess cognitive functions in diverse neurological and psychiatric disorders, such as dementia, schizophrenia, and depression. The tests of CANTAB are independent of culture and measure several neurocognitive functions and domains including visual memory, executive function, attention, semantic/verbal memory, decision making and social cognition. The Hungarian version was first described by Bartók et al. in 2001 [48]. The first two tests (Motor screening, MOT, and Big-Little Circle, BLC) were used as screening tests to assess the subjects’ mental and physical suitability for the examination procedure. After these, the following tests were administered: Reaction Time (RTI), Intra-Extradimensional Shifting (IED), Rapid Visual Information Processing (RVP), Stockings of Cambridge (SOC), Spatial Working Memory (SWM), Spatial Span (SSP), Paired Associates Learning (PAL), and Cambridge Gambling Test (CGT) (Table 2). These tests were selected on the basis of previous results showing their sensitivity in ADHD, and the availability of the tests in the Hungarian version of CANTAB.

**Table 2:** Classification of the used CANTAB subtests according to key domains and cognitive processes

Key domain	Cognitive processes	CANTAB subtests
Attention and psychomotor speed	Motor speed and cognitive speed/reaction time	Reaction time (RTI)
	Sustained attention	Rapid visual information processing (RVP)
	Impulsivity (impulsive response)	Reaction time (RTI) Rapid visual information processing (RVP)
Memory	Visual memory and learning	Paired associates learning (PAL)
	Visuospatial working memory capacity	Spatial span (SSP)
Executive functions	Retention and manipulation of visuospatial information	Spatial working memory (SWM)
	Mental flexibility	Intra-extra dimensional set shift (IED)
	Spatial planning	Stocking of Cambridge (SOC)
	Decision making and risk taking behavior	Cambridge gambling task (CGT)

### Statistical procedures

Statistical analyses were performed using SPSS version 22.0 (SPSS Inc., Chicago, Illinois, USA) [49] and SAS 9.4. (SAS Institute, Cary NC) [50]. Comparison of continuous and categorical demographic variables was carried out by the independent samples *t*-test and Chi-square test, respectively. The comparison of CANTAB variables between two (aADHD and HC) or three (aADHD subtypes and HC) groups was performed in two steps: first, raw data were used, and generalized linear model (GLM) analyses were performed, with gender, age and education level considered as confounders. The FDR method was applied to correct for multiple comparisons. To compare effect sizes of cognitive performance measures between the aADHD and HC groups, Cohen-*d* values were calculated, which were used to rank output variables.

Since neurocognitive variables measured with CANTAB are not independent from each other, we combined a factor-analytic approach including multiple CANTAB variables for each task and multivariate pattern analysis, with the aim of data-reduction. This multivariate approach complements the above described univariate comparisons. Factor analysis with varimax rotation was used on all variables except for BLC, MOT and SSP, factors with an Eigenvalue above 1 were retained [51]. CANTAB variables with an absolute factor loading over 0.7 were identified, and were assigned to one of the factors based on the highest factor loading. Variables assigned to the same factor were collapsed together to a canonical variable. Next, we conducted multi-group confirmatory factor analysis (MGCFA) to assess whether the structure of latent cognitive traits was similar in patients and controls [52–54].

These canonical variables served as input to stepwise discriminant models to find the most significant factors that differentiate study groups. During this multivariate pattern-analysis the forward selection begins with no variables in the model. At each step, the model enters the variable that contributes most to the discriminatory power of the model as measured by Wilks’s lambda, the likelihood ratio criterion. When none of the unselected variables meet the entry criterion, the forward selection process stops. Finally, to explore possible associations between neurocognitive variables and subjective symptom severity, Pearson’s correlations were calculated between canonical factors and CAARS subscores in the aADHD sample.

## Results

### Clinical and demographic data

Basic demographic and clinical characteristics of the aADHD and HC groups are demonstrated in Table 1. The groups were comparable in sex, age and educational level. Assessment of comorbidities revealed that more than half of the ADHD patients had at least one other psychiatric diagnosis, most of them suffering from major depressive disorder and anxiety disorders. The aADHD group had higher severity of general psychopathology in all variables, as measured by the SCL-90-R scale, and as expected, patients displayed higher severity on all CAARS symptom dimensions, including inattention, hyperactivity, impulsivity, problems with self-concept, and on all specific, DSM-based symptom scales. Mean IQ score of the aADHD group was 125.8 (SD = 12.12).

### Comparison of neurocognitive performance between the aADHD and HC groups

**Fig. 1:**
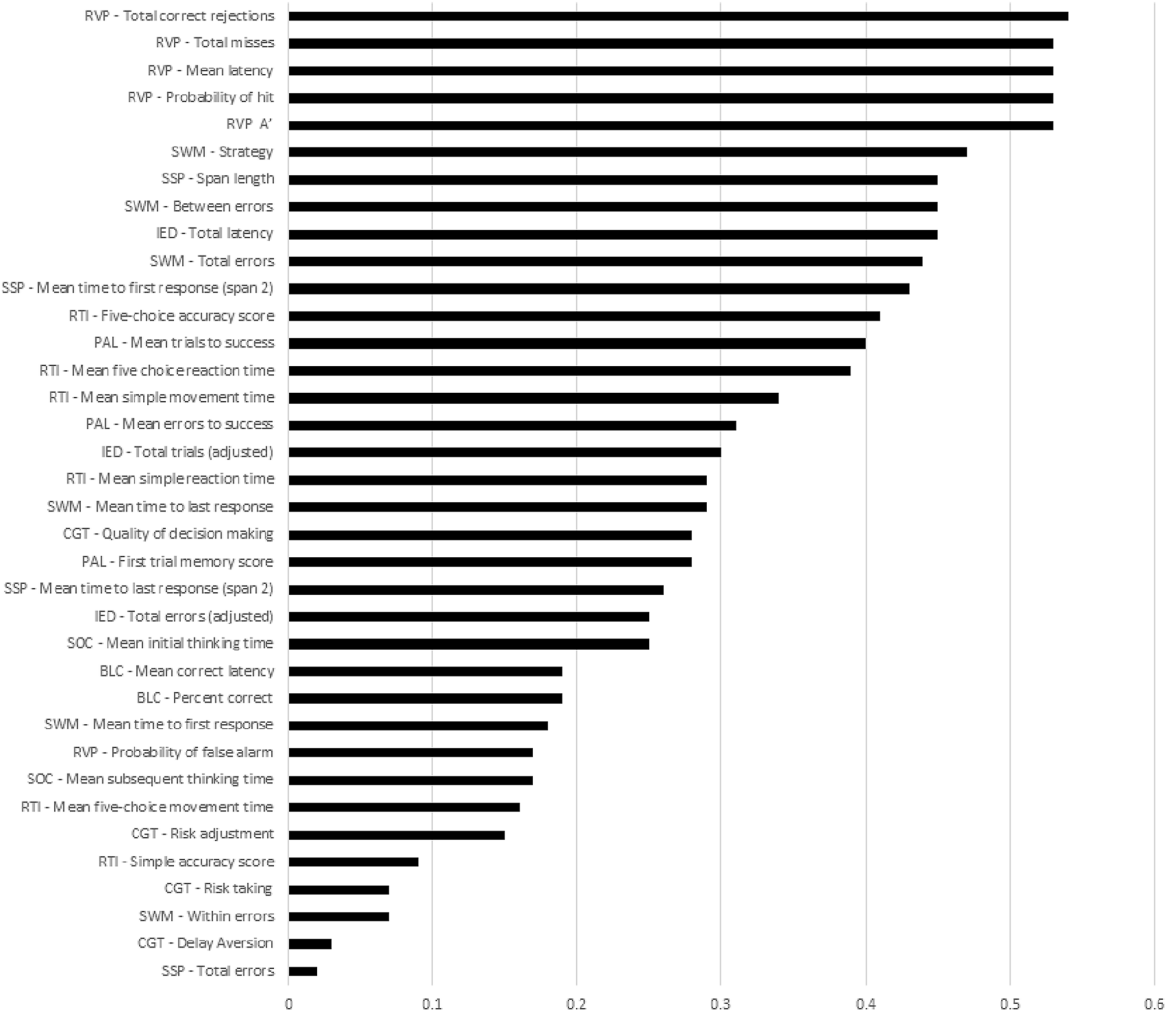
Cohen-*d* values of the most important CANTAB variables based on comparisons between the aADHD and HC groups in the order of absolute values *RVP* rapid visual information processing, *IED* intra/extradimensional shifting, *SWM* spatial working memory, *SSP* spatial span, *RTI* reaction time, *PAL* paired association learning, *BLC* big/little circle, *CGT* Cambridge gambling task

First, we made comparisons between the aADHD and the healthy control groups’ cognitive performance measured by the CANTAB battery (Table 3). These results showed decreased cognitive performance in the aADHD group impacting the following domains and task variables: sustained attention (RVP, RVP A′, Probability of hit, Mean latency, Total correct rejection, Total misses), spatial working memory (SWM, Strategy, Total errors, Between errors), working memory capacity (SSP, Span length, Total errors), visual memory (PAL, Mean trials to success), reaction time (RTI, Five-Choice reaction time, Five-choice accuracy score), and the latency measure of the cognitive flexibility/set-shifting task (IED, Total latency). The between-group differences in these variables were nominally significant, resulting in moderate effect sizes with Cohen-*d* values between 0.38 and 0.62 (Fig. 1), but only the sustained attention domain RVP-variables survived FDR-correction for multiple comparisons (*p* = 0.037). We found no differences between groups in other variables.

**Table 3:** Raw neuropsychological variables and GLM comparison between the aADHD and HC groups

CANTAB test	aADHD	HC	F	*p*	FDR-corr. (*p*)	Effect size (Cohen’s-*d*)
*Big little circle*^*n*^^1^						
Percent correct	99.59 (0.16)	99.83 (0.16)	1.04	0.3092	0.4281	0.19
Mean correct latency	883.25 (29.8)	837.95 (30.55)	1.08	0.3008	0.4281	− 0.19
*Stocking of Cambridge*^*n*^^1^						
Mean initial thinking time	9128.95 (1092)	11,252.95 (1124.11)	1.79	0.1832	0.2748	0.25
Mean subsequent thinking time	999.81 (162.35)	789.66 (166.48)	0.83	0.3635	0.4513	− 0.17
*Intra/extradimensional shifting*^*n*^^1^						
Total errors (adjusted)	16.47 (1.24)	14.04 (1.27)	1.84	0.1777	0.2748	− 0.25
Total latency	132,764.13 (7437)	106,435 (7651)	6.07	**0.0152**	0.0693	− 0.45
Total trials (adjusted)	80.56 (2.2)	75.32 (2.26)	2.68	0.1041	0.2204	− 0.30
*Rapid visual information processing*^*n*^^1^						
RVP A′	0.9 (0.01)	0.93 (0.01)	8.15	**0.0051**	**0.0368**	0.53
Probability of false alarm	0.01 (0.001)	0 (0.001)	0.85	0.3586	0.4513	− 0.17
Probability of hit	0.63 (0.02)	0.73 (0.02)	8.32	**0.0047**	**0.0368**	0.53
Mean latency	483.35 (16.58)	414.41 (17.04)	8.25	**0.0049**	**0.0368**	− 0.53
Total correct rejections	249.72 (1.48)	255.97 (1.52)	8.46	**0.0044**	**0.0368**	0.54
Total misses	9.98 (0.65)	7.26 (0.67)	8.31	**0.0047**	**0.0368**	− 0.53
*Spatial working memory*^*n*^^1^						
Strategy	31.69 (0.8)	28.69 (0.82)	6.62	**0.0114**	0.0683	− 0.47
Mean time to first response	2394.49 (144.62)	2186.07 (147.86)	0.96	0.3286	0.4382	− 0.18
Mean time to last response	28,339 (894.5)	26,316.3 (920.3)	2.43	0.1216	0.2246	− 0.29
Total errors	24.08 (2.19)	16.54 (2.24)	5.63	**0.0193**	0.0696	− 0.44
Within errors	1.89 (0.42)	1.67 (0.44)	0.14	0.7128	0.7619	− 0.07
Between errors	23.14 (2.14)	15.64 (2.19)	5.83	**0.0173**	0.0693	− 0.45
*Paired association learning*^*n*^^1^						
First trial memory score	20.59 (0.49)	21.66 (0.5)	2.31	0.1310	0.2246	0.28
Mean errors to success	1.19 (0.16)	0.8 (0.17)	2.83	0.0951	0.2140	− 0.31
Mean trials to success	1.46 (0.05)	1.3 (0.05)	4.81	**0.0303**	0.0838	− 0.40
*Cambridge gambling task*^*n*2^						
Delay aversion	0.2 (0.03)	0.21 (0.03)	0.03	0.8736	0.8985	0.03
Quality of decision making	0.94 (0.01)	0.96 (0.01)	2.35	0.1280	0.2246	0.28
Risk adjustment	1.52 (0.15)	1.69 (0.15)	0.70	0.4034	0.4684	0.15
Risk taking	0.58 (0.02)	0.57 (0.02)	0.13	0.7196	0.7619	− 0.07
*Reaction time*^*n*3^						
Mean five-choice movement time	462.99 (18.75)	439.52 (19.32)	0.76	0.3849	0.4619	− 0.16
Mean five choice reaction time	369.95 (9.69)	340.71 (9.98)	4.42	**0.0379**	0.0974	− 0.39
Simple accuracy score	8.8 (0.06)	8.84 (0.06)	0.23	0.6348	0.7141	0.09
Mean simple movement time	542.79 (25.41)	475.85 (26.11)	3.38	0.0685	0.1645	− 0.34
Mean simple reaction time	359.02 (11.55)	332.85 (11.76)	2.54	0.1140	0.2246	− 0.29
Five-choice accuracy score	0.07 (0.02)	0 (0.02)	5.04	**0.0267**	0.0800	− 0.41
*Spatial span*^*n*4^						
Span length	6.42 (0.19)	7.1 (0.18)	5.38	**0.0224**	0.0732	0.45
Mean time to first response (span 2)	2803.69 (98.43)	2495.16 (89.3)	1.94	0.1670	0.2733	− 0.43
Mean time to last response (span 2)	3776.41 (122.07)	3548.6 (110.61)	0.02	0.8993	0.8993	− 0.26
Total errors	13.7 (1.04)	13.88 (0.95)	5.98	**0.0162**	0.0693	0.02

Variables are presented as least square means (mean adjusted with age, gender and education level by the GLM) and standard error values. The values represent percent, time measured in milliseconds, or numbers. GLM comparison were carried out between the aADHD and HC groups, uncorrected and FDR-corrected *p*-values are presented in conjunction with effect sizes

Statistically significant results with a *p*-value under 0.05 are highlighted in bold

^*n*^^1^*N* = 119; ^*n*2^*N* = 116; ^*n*3^*N* = 117; ^*n*4^*N* = 105

### Comparison of neurocognitive performance between aADHD subtypes

Next, we performed comparison between the aADHD subgroups and healthy controls (HC) using GLM analyses. The subgrouping was based on the presence of DSM-5 hyperactive-impulsive threshold. Because of the small number of purely hyperactive-impulsive patients, we investigated two patient subgroups, the inattentive group (aADHD-I) and the mixed group (aADHD-MIX) consisting of aADHD-HI and aADHD-C patients. The between-subtype comparisons revealed differences reaching nominal significance in several cognitive domains and CANTAB test variables (Table 4). The affected domains were sustained attention (RVP A′, probability of hit, total correct rejections, total misses, mean latency), working memory (SWM, strategy, total errors, between errors, SSP, span length, mean time for first response), and cognitive flexibility (IED, total latency). Post-hoc analyses showed decreased cognitive performance in aADHD-I compared to HC in RVP, SWM, and CGT variables. Comparisons between aADHD-MIX and HC groups yielded nominally significant results in the IED, SWM, RVP, RTI and SSP tests. None of these between-subtype differences survived FDR correction for multiple comparisons.

**Table 4:** Raw neuropsychological variables and GLM comparison between aADHD subtypes and HC group with post-hoc analyses

CANTAB variable	ADHD-I	ADHD-MIX	HC	*F*	*p*	FDR-corr. (*p*)	HC-ADHD-I	HC-MIX	ADHD-I-MIX
*Big little circle*^*n*^^1^									
Percent correct	99.53 (0.21)	99.64 (0.25)	99.83 (0.16)	0.58	0.5630	0.6989	0.3164	0.4893	0.7302
Mean correct latency	920.45 (39.81)	854.79 (45.61)	837.95 (30.55)	1.12	0.3296	0.4736	0.1398	0.7375	0.2838
*Stocking of Cambridge*^*n*^^1^									
Mean initial thinking time	8310.87 (1464)	9754.78 (1677)	11,252.95 (1124.11)	1.10	0.3367	0.4736	0.1522	0.4181	0.5211
Mean subsequent thinking time	799.44 (216.93)	1153.1 (248.5)	789.66 (166.48)	0.98	0.3772	0.4850	0.9743	0.1858	0.2893
*Intra/extradimensional shifting*^*n*^^1^									
Total errors (adjusted)	17.31 (1.66)	15.83 (1.9)	14.04 (1.27)	1.08	0.3420	0.4736	0.1600	0.3920	0.5625
Total latency	127,373 (9970)	136,887 (11,421)	106,435 (7651)	3.21	**0.0440**	0.1549	0.1346	**0.0168**	0.5345
Total trials (adjusted)	82.73 (2.95)	78.91 (3.38)	75.32 (2.26)	1.70	0.1880	0.3561	0.0742	0.3361	0.3995
*Rapid visual information processing*^*n*^^1^									
RVP A′	0.9 (0.01)	0.91 (0.01)	0.93 (0.01)	4.60	**0.0120**	0.0978	**0.0051**	0.0572	0.3104
Probability of false alarm	0.01 (0.001)	0.01 (0.001)	0 (0.001)	0.42	0.6572	0.7430	0.4867	0.4275	0.9844
Probability of hit	0.6 (0.03)	0.65 (0.04)	0.73 (0.02)	4.67	**0.0113**	0.0978	**0.0049**	0.0534	0.3152
Mean latency	499.54 (22.2)	470.96 (25.43)	414.41 (17.04)	4.47	**0.0136**	0.0978	**0.0069**	**0.0453**	0.4023
Total correct rejections	248.09 (1.98)	250.96 (2.27)	255.97 (1.52)	4.67	**0.0112**	0.0978	**0.0052**	**0.0475**	0.3466
Total misses	10.73 (1.00)	9.41 (0.87)	7.26 (0.67)	4.64	**0.0115**	0.0978	0.0525	**0.0051**	0.3248
*Spatial working memory*^*n*^^1^									
Strategy	32.54 (1.07)	31.03 (1.23)	28.69 (0.82)	3.72	**0.0272**	0.1259	**0.0115**	0.0863	0.3615
Mean time to first response	2619.35 (192.67)	2222.46 (220.71)	2186.07 (147.86)	1.39	0.2535	0.4147	0.1094	0.8810	0.1813
Mean time to last response	28,984.87 (1199.27)	27,845.04 (1373.8)	26,316.34 (920.38)	1.40	0.2503	0.4147	0.1131	0.3134	0.5361
Total errors	27.39 (2.92)	21.54 (3.34)	16.54 (2.24)	3.69	**0.0280**	0.1259	**0.0086**	0.1754	0.1926
Within errors	1.93 (0.57)	1.86 (0.65)	1.67 (0.44)	0.07	0.9320	0.9627	0.7398	0.7836	0.9390
Between errors	26.37 (2.85)	20.67 (3.26)	15.64 (2.19)	3.79	**0.0255**	0.1259	**0.0079**	0.1639	0.1934
*Paired association learning*^*n*^^1^									
First trial memory score	20.68 (0.65)	20.52 (0.75)	21.66 (0.5)	1.16	0.3176	0.4736	0.2852	0.1710	0.8770
Mean errors to success	1.3 (0.22)	1.11 (0.25)	0.8 (0.17)	1.56	0.2137	0.3847	0.1011	0.2530	0.5766
Mean trials to success	1.49 (0.07)	1.43 (0.08)	1.3 (0.05)	2.52	0.0851	0.2162	**0.0466**	0.1094	0.6151
*Cambridge gambling task*^*n*2^									
Delay aversion	0.23 (0.03)	0.18 (0.04)	0.21 (0.03)	0.44	0.6429	0.7430	0.6196	0.5515	0.3553
Quality of decision making	0.92 (0.01)	0.95 (0.02)	0.96 (0.01)	2.43	0.0926	0.2162	**0.0299**	0.5744	0.1180
Risk adjustment	1.54 (0.19)	1.51 (0.23)	1.69 (0.15)	0.35	0.7027	0.7666	0.5795	0.4390	0.9184
Risk taking	0.58 (0.02)	0.57 (0.03)	0.57 (0.02)	0.07	0.9359	0.9627	0.7568	0.7802	0.9488
*Reaction time*^*n*3^									
Mean five-choice movement time	456.78 (25)	467.59 (29.14)	439.52 (19.32)	0.42	0.6605	0.7430	0.6257	0.3757	0.7805
Mean five choice reaction time	362.75 (12.91)	375.28 (15.04)	340.71 (9.98)	2.39	0.0961	0.2162	0.2290	**0.0361**	0.5316
Simple accuracy score	8.89 (0.08)	8.73 (0.09)	8.84 (0.06)	1.02	0.3654	0.4850	0.6241	0.2750	0.1821
Mean simple movement time	517.47 (33.79)	561.52 (39.37)	475.85 (26.11)	2.04	0.1347	0.2852	0.3845	**0.0469**	0.4013
Mean simple reaction time	336.18 (15.21)	375.91 (17.73)	332.85 (11.76)	2.71	0.0707	0.1957	0.8771	**0.0269**	0.0942
Five-choice accuracy score	0.05 (0.03)	0.08 (0.04)	0 (0.02)	2.74	0.0689	0.1957	0.2019	**0.0250**	0.4990
*Spatial span*^*n*4^									
Span length	6.29 (0.31)	6.54 (0.27)	7.1 (0.18)	3.14	**0.0473**	0.1549	0.0966	**0.0266**	0.5568
Mean time to first response (span length 2)	2680.68 (136.34)	2910.24 (146.83)	2495.16 (89.3)	3.33	**0.0396**	0.1549	0.2901	**0.0122**	0.2621
Mean time to last response (span length 2)	3609.94 (168.88)	3920.59 (181.87)	3548.6 (110.61)	1.73	0.1821	0.3561	0.7770	0.0676	0.2208
Total errors	13.74 (1.45)	13.68 (1.56)	13.88 (0.95)	0.01	0.9917	0.9917	0.9369	0.9050	0.9781

Variables are presented as least square means (mean adjusted with age, gender and education level by the GLM) and standard error values. The values represent percent, time measured in milliseconds, or numbers. GLM comparison were carried out between aADHD subtypes and the HC groups, uncorrected and FDR-corrected *p*-values are presented in conjunction with post-hoc *p*-values

Statistically significant results with a *p*-value under 0.05 are highlighted in bold

^*n*^^1^*N* = 119; ^*n*2^*N* = 116; ^*n*3^*N* = 117; ^*n*4^*N* = 105

### Multivariate pattern analysis based on principal component and step-wise discriminant analysis

**Fig. 2:**
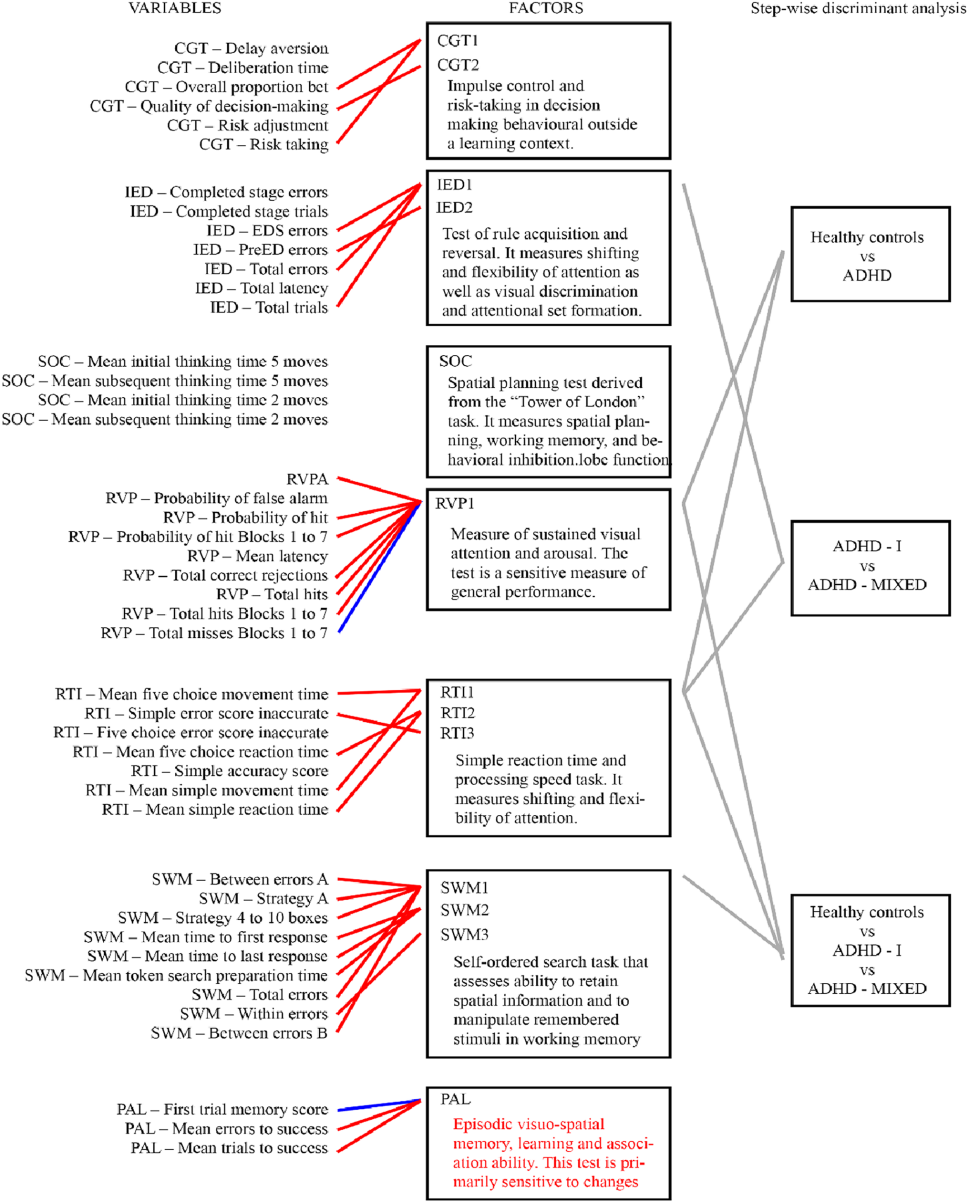
Summary of principal component and multivariate pattern analysis procedures. The CANTAB variables used in the multivariate pattern analysis are shown on the left. Colored lines represent factor absolute loadings above 0.7 (red indicates positive loads, while blue indicates negative loads) to the 12 factors presented in the middle column. Short descriptions of factors are presented under factor abbreviations. Finally, connections between the factors and the 3 separate step-wise discriminant analyses are highlighted on the right side. *RVP* rapid visual information processing, *IED* intra/extradimensional shifting, *SWM* spatial working memory, *SSP* spatial span, *RTI* reaction time, *PAL* paired association learning, *BLC* big/little circle, *CGT* Cambridge gambling task

As a data-reduction technique, we used principal component analysis. Factor analysis of 46 variables yielded 12 factors with an eigenvalue higher than one. Thirty-one variables were assigned to one of the factors based on the absolute value of factor loadings above 0.7 (Table 5). The identified factors separated exclusively according to CANTAB tests, i.e., we found no factor loaded by different CANTAB subtests. Four of these factors were excluded from downstream analyses because they were only loaded by one variable. Multi-group confirmatory factor analysis performed as a quality-control step suggested 11 factors as optimal. The structure of three factors were questioned at this quality-control step. The IED1 factor was also loaded by “IED Completed stage trials and errors” in both the aADHD and control groups. The SWM2 factor was also loaded with “SOC Mean initial thinking time 5 moves” in the HC group, and the RTI1 “Mean simple movement time” was replaced with “SWM Within errors” variable in the aADHD group. Figure 2 summarizes results of the multivariate pattern-analysis procedures.

**Table 5:** Factors identified using principal component analysis, corresponding load factors with scores, and short descriptions

Factor names	CANTAB variable	Loading score	Description
RVP1	RVP A′	0.94	Ability to focus on target and other sustained attentional measures
	RVP probability of hit	0.93	
	RVP probability of hit blocks 1 to 7	0.92	
	RVP total correct rejections	0.93	
	RVP total hits	0.93	
	RVP total hits blocks 1 to 7	0.92	
	RVP total misses blocks 1 to 7	− 0.92	
SWM1	SWM between errors A	0.84	Ability to retain spatial information and manipulate remembered items in working memory. Efficient strategy for completing the task
	SWM strategy	0.82	
	SWM strategy 4 to 10 boxes	0.83	
	SWM total errors	0.83	
	SWM between errors	0.84	
IED1	IED EDS errors	0.83	Cognitive flexibility
	IED total errors	0.89	
	IED total trials	0.88	
SWM2	SWM mean time to first response	0.85	Task solving speed in working memory test
	SWM mean time to a st. response	0.81	
	SWM mean token search prep. time	0.92	
PAL	PAL first trial memory score	− 0.81	Visual memory
	PAL mean errors to success	0.87	
	PAL mean trials to success	0.84	
CGT1	CGT overall proportion bet	0.95	Self-control measures in gambling task
	CGT risk taking	0.95	
RTI1	RTI mean five choice move time	0.91	Movement time measures (motor functions)
	RTI mean simple movement time	0.88	
IED2	IED pre ED errors	0.79	The number of errors made prior to the extra-dimensional shift of the task
RTI2	RTI mean five choice reaction time	0.85	Reaction time
	RTI mean simple reaction time	0.80	
RTI3	RTI simple error score inaccurate	0.74	Inaccurate errors meaning, that the subject does not touch the circle with sufficient position
CGT2	CGT quality of decision making	0.80	This measure is the proportion of trials where the subject chose the more likely outcome
SWM3	SWM within errors	0.72	Within errors are defined as the number of errors made within a search

**Fig. 3:**
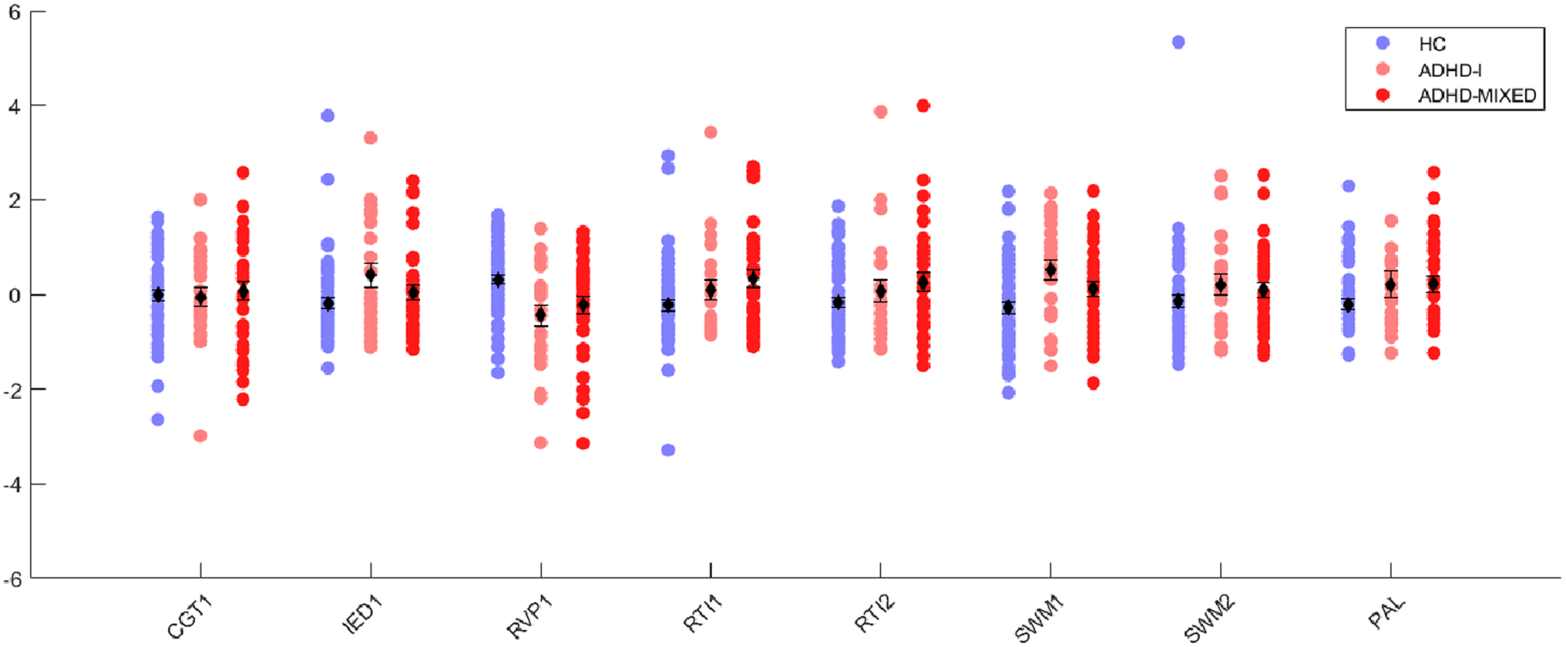
Canonical variables, derived for factors loaded by more than one variable, are illustrated for aADHD subtypes and the HC group. Circles denote for each subject in the corresponding groups, black error bars represent group means

Comparison of the remaining 8 canonical variables demonstrated significant differences between aADHD and HC groups in the RVP1 and SWM1 factors (*p* < 0.02 after FDR correction), representing the domains of sustained visual attention and spatial working memory (Fig. 3 and Table 6). Canonical variables derived for each factor were used for performing stepwise discriminant analysis. First, healthy controls and the aADHD group were best separable with 2 factors, namely RVP1 and RTI1. Next, the discrimination between all the three groups was tested resulting in SWM1, RVP1 and RTI1 as discriminating factors. Finally, we repeated the analysis to identify factors that could differentiate between aADHD subtypes most efficiently. This analysis yielded 2 discriminating factors, RTI1 and IED1 (Table 7 and Supplementary Table 1).

**Table 6:** GLM comparison of canonical variables between aADHD and HC groups, with FDR-corrected *p*-values

Factor name	Three-group comparisons						Two-group comparisons		
	*F*	*p*	FDR (*p*)	HC-ADHD-I	HC-ADHD-MIX	ADHD-I-ADHD-MIX	*F*	*p*	FDR (*p*)
CGT1	0.10	0.9027	0.9108	0.9823	0.6748	0.7245	0.08	0.7768	0.8474
IED1	2.42	0.0935	0.1493	0.0325	0.2882	0.2543	3.52	0.0633	0.1265
RVP1	**6.33**	**0.0025**	**0.0192**	**0.0019**	**0.0124**	0.3670	**11.85**	**0.0008**	**0.0098**
RTI1	**3.65**	**0.0293**	0.1171	0.2975	**0.0082**	0.2443	**5.91**	**0.0167**	0.0501
RTI2	2.60	0.0791	0.1493	0.3455	0.0253	0.3574	**4.35**	**0.0394**	0.0946
SWM1	**6.06**	**0.0032**	**0.0192**	**0.0012**	**0.0467**	0.1432	**9.83**	**0.0022**	**0.0132**
SWM2	1.40	0.2519	0.3358	0.1211	0.2886	0.5553	2.46	0.1199	0.1798
PAL	3.01	0.0534	0.1493	0.0891	0.0288	0.8600	**6.04**	**0.0155**	0.0501

Statistically significant results with a *p*-value under 0.05 are highlighted in bold

**Table 7:** Results of step-wise discriminant analyses between 2 and 3 groups

Healthy control vs. aADHD^a^		
Factor names	*F*	*p*
RVP1	11.16	**0.0011**
RTI1	2.58	0.1109

Healthy control vs. ADHD-inattentive vs. ADHD-MIX^b^		
Factor names	*F*	*p*
RVP1	6.24	**0.0027**
RTI1	2.35	0.0997
SWM1	2.22	0.1133

ADHD-inattentive vs. ADHD-MIX^c^		
Factor names	*F*	*p*
RTI1	2.18	0.1460
IED1	2.93	0.0928

Statistically significant results with a *p*-value under 0.05 are highlighted in bold

^a^Two groups comparison in whole ADHD and healthy control group (HC)

^b^Three groups comparison of healthy control, inattentive ADHD and mixed ADHD (hyperactive-impulsive and combined types) groups

^c^Two groups comparison of ADHD groups

### Correlations between CANTAB-based canonical variables and self-reported symptom severity

**Fig. 4:**
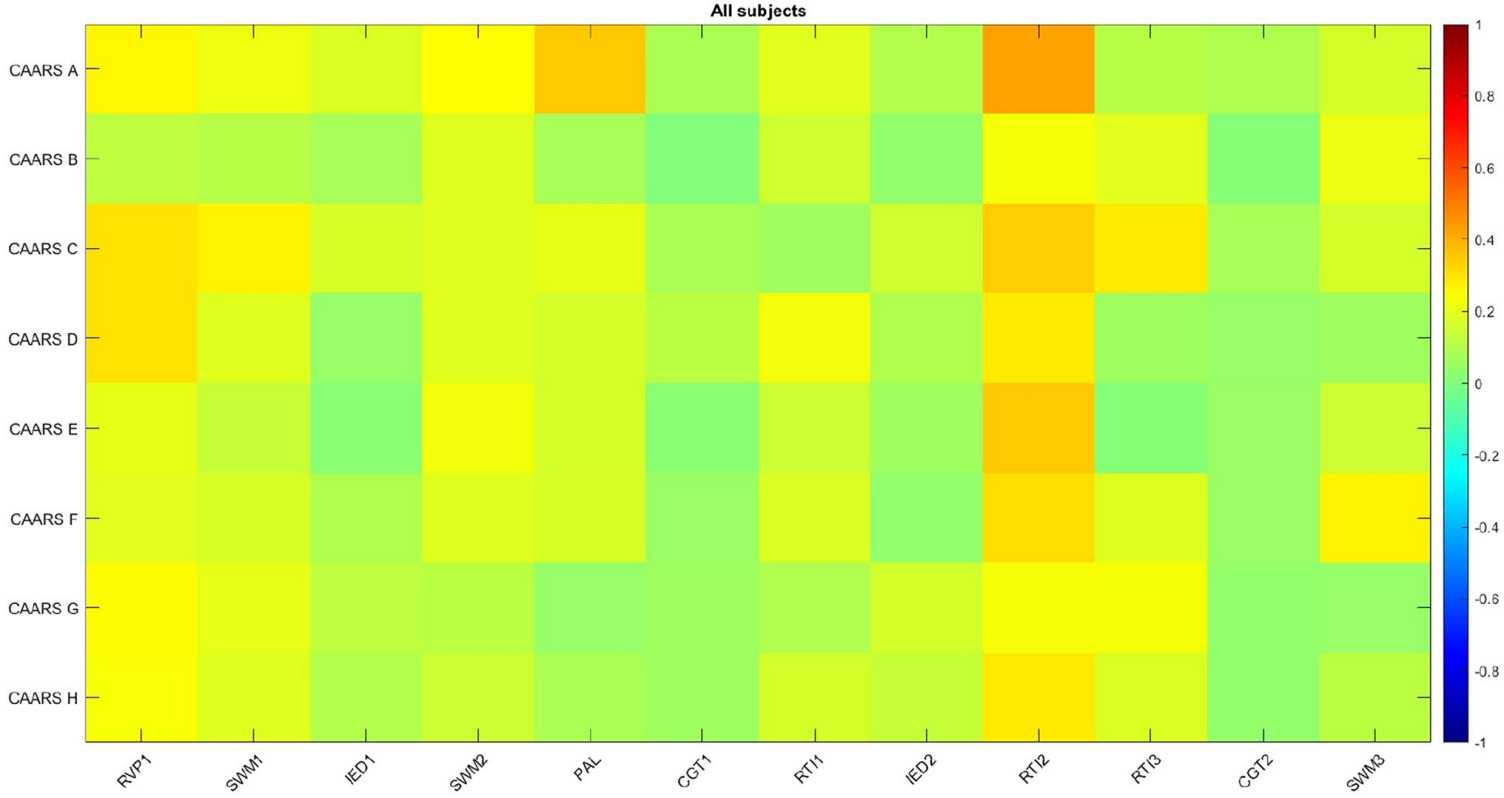
Heatmap demonstrating correlations of self-reported symptom severity measured by CAARS and neurocognition factors in the whole sample. Hot colors show positive, while cold colors indicate negative correlation. Colors are scaled between − 1 and 1. Rows represent psychopathology measures with CAARS subscores and columns represent factor based canonical variables

Finally, correlation analyses were carried out between the self-reported symptom severity variables measured by CAARS, and the cognitive domains based on canonical variables. We found only scattered correlations demonstrating nominal statistical significance, with weak to moderate correlation coefficients. After correction for sign (necessary because of the inverse scoring in this test), the RVP1 factor showed correlations with the CAARS-C and CAARD-D subscales, the PAL factor was correlated with CAARS-A, while the RTI2 factor had the most positive correlations with different CAARS subscales. An overview of correlation coefficients is presented as a heatmap in Fig. 4.

## Discussion

This study aimed to compare neuropsychological functioning between groups of adults living with ADHD and healthy controls, as well as between aADHD subtypes. Moreover, we sought to investigate the relationship between self-reported symptom severity and neurocognition in a thoroughly characterized clinical sample. To our knowledge, the current study is the first to use CANTAB to examine the neuropsychological characteristics of ADHD in a sample of Hungarian adults, a country representing Eastern-Central Europe, a region where aADHD is still underdiagnosed compared to Western European countries, due to the distinct developmental course of the mental health care system during past decades.

Comparison of adults diagnosed with ADHD to matched healthy controls yielded significant differences in certain CANTAB subtests indicative of decreased performance in attention and psychomotor speed (sustained visual attention, signal detectability, alertness, processing speed, shifting and flexibility of attention), executive functions (spatial working memory), and memory (short-term spatial memory, visual memory). In domains of higher executive functions (spatial planning and problem solving, decision making and risk-taking behavior) we could not observe differences between the two groups. It is important to note that after FDR-correction only the RVP subtest variables measuring sustained visual attention, alertness, and signal detectability remained significant.

It is conceivable that the significant differences at the RVP subtest reflect decreased sustained attention capacity, which aADHD patients are not able to improve by compensation and adaptation strategies, due to the unique characteristics of this test, such as time-pressure and duration. While compensation strategies, such as increased focusing may improve performance in other subtests, they have a very limited effect in the RVP subtest. In line with this interpretation both two- and three-group comparisons in our sample indicated increased mean latency and total miss values in the aADHD groups compared to the HC group. Indeed, sustained attention has been shown to be the most sensitive marker of vigilance dysfunction in different modalities, including neuropsychological [55, 56], functional brain imaging [57], and electrophysiology studies [58]. It has been suggested that deficits in sustained attention potentially reflect catecholamine (dopamine and norepinephrine) dysregulation [59, 60]. In the other tests (SWM, SSP, PAL, IED) only nominally significant differences were detectable, which did not survive FDR correction. These less pronounced differences can be explained by cognitive compensation strategies, which are typical for adults with ADHD, but less developed and observable in childhood ADHD [61]. Overall, in line with previous studies [27], our findings show that neuropsychological testing with the CANTAB software is helpful at identifying deficits in individual patients at specific cognitive domains like attention, executive function, memory, but cannot provide a clear diagnostic reference point and cutoff scores to properly separate patients and healthy controls. Based on our results, the RVP test is the most reliable for discriminating the two groups.

Neurocognitive heterogeneity has been shown not only across, but also within studies of aADHD [62], therefore it seems essential to investigate whether there are differences in cognitive performance between aADHD symptom-based subtypes. According to our results, the performance of the aADHD-I and aADHD-MIX groups are both significantly different when compared to healthy controls, but the implicated functions are divergent. Overall, adults with predominantly inattentive symptoms showed more deviations compared to matched controls than individuals with mixed symptoms, namely difficulties in spatial working memory (SWM, strategy, total and between errors) and visual memory (PAL, mean trials to success). In comparison, adults with mixed symptoms showed difficulties in short-term spatial memory, and both aADHD groups showed poorer performance in sustained attention and signal detectability variables (RVP). Mean latency was increased in both subtypes, however in aADHD-MIX patients this was paralleled with better accuracy (probability of hit) and lower number of total misses, despite the impulsive symptoms. The pattern of alterations can be interpreted as a partially successful compensation strategy of the aADHD-MIX individuals. These findings, although based on only nominally significant differences, support the hypothesis that individuals with aADHD-I can be affected more severely by neurocognitive deficits. In children, results remain diverse in this regard [28, 29].

It is an interesting question why cognitive performance is not affected as severely in the aADHD-MIX group where attention deficit is also present at the symptomatic level, and why results do not reflect the symptoms of impulsivity and hyperactivity. For example, response inhibition measured by probability of false alarm, commission errors in the RTI task, or alterations of decision making in the CGT tasks were unchanged, however RVP mean latency was affected in the aADHD-MIX group as well. We can hypothesize that motor impulsivity is better compensated in the combined subtype, and subjective attentional problems can be the results of secondary phenomena associated with increased compensation demand and impulsive traits.

Previous studies exploring the structure of CANTAB variables with a factor-analytic approach used only a limited number of CANTAB variables, investigating usually only one raw variable per task [63, 64]. Since multiple variables may characterize specific aspects of the subtest, we carried out a multivariate pattern analysis on multiple CANTAB variables. Multi-group confirmatory factor analysis showed preserved factor structure in both groups compared to pooled subjects. Using stepwise discriminant analysis differentiation between aADHD and controls was best among factors measuring sustained visual attention, signal detectability and arousal (RVP1) and psychomotor speed (RTI1), while separating three groups was best achieved with the inclusion of the spatial working memory (SWM1) factor to the above factors. The two aADHD subtypes were best separable with RTI1 and a factor measuring primarily set-shifting and flexibility of attention (IED1). The combination of RVP1 and RTI1 can be interpreted as sustained attention deficit and emergent compensatory mechanisms, moreover, the differences at IED1 between aADHD subtypes represent the deficits of cognitive flexibility necessary to dynamically adjust attention and behavior in various situations by monitoring and compensating performance [65, 66]. As expected, the multivariate comparisons applying data-reduction techniques yielded more significant results between groups, than head-to-head comparisons of raw neuropsychological variables. For example, both two- and three-group comparisons demonstrated differences both at the RVP1 and SWM1 factors, while in head-to-head comparisons only RVP variables survived correction for multiple comparisons.

Correlations between DSM-based symptom severity and performance on neuropsychological tests, as reflected by factors were generally low. Our findings showed that higher inattention and memory problems in the CAARS assessment scores were related to slower reaction time and difficulties in shifting, reflected by higher scores of the RTI2 factor. In addition, higher levels of impulsivity, emotional lability and problems with self-concept reported in CAARS scores were associated with worse sustained visual attention, signal detectability and arousal, reflected by lower scores of the RVP1 factor. No other significant correlations were found. The present results are in line with findings from previous studies [31], demonstrating low levels of correlation between self-reported complaints and objective neurocognitive performance. Adding to the observed heterogeneity, it has been shown recently that late-onset aADHD patients have a distinct neurocognitive profile with less impairment in alertness and executive functioning [67]. These findings support the hypothesis that cognitive profile can be more closely related to functional impairments than to symptoms per se [62]. The differences between the aADHD and HC groups in the subjective and neuropsychological tests as well, and the fact that they were loosely associated, suggests that both are necessary during the assessment of symptoms, these screening methods are not interchangeable. According to a recent study conducted in childhood ADHD [68], while children with mixed symptoms had more behavioral problems and emotional lability, the ADHD-I group showed more impairment in sustained attention and higher degree of brain white matter alterations.

Our findings should be interpreted with the acknowledgment of some strengths and limitations. It is important that all the patients had a valid ADHD diagnosis and were examined for potential comorbidities according to DSM-5 by experienced clinical professionals. These steps are crucial to get a more accurate picture and were often limitations in previous research [22]. It is important to note that the intelligence level is relatively high in our patient sample, while IQ scores for healthy controls was not available. The high average IQ score in patients suggests that the generalizability of our results is limited and the possibility of compensation strategies is also plausible, a feature that is both a strength and limitation of the study. The statistical approach was designed to control for multivariate effects. Unfortunately, contrary to recommendations, we did not have the opportunity to use Stop Signal Task (which measures response inhibition), because of language limitations at the time of data-acquisition, which is a limitation of the study. Although CANTAB has many advantages, among others, culture-independency, standardized, computerized measurement, assessment of several cognitive domains, it should be emphasized that it relates mainly to spatial-visual functions and we do not have information about other modalities. It is worth mentioning that some people with ADHD do not show any significant deficits compared to controls, that is why future studies should target the individual’s cognitive profile, rather than looking at differences from the average. Furthermore, a major challenge for the future can be to develop more ecologically valid test conditions that better model everyday life and the many stimuli we face while trying to perform efficiently. In addition, future work would benefit of including a predominantly hyperactive-impulsive subgroup to better understand the differences in terms of neurocognitive alterations between aADHD subtypes.

In summary, living with ADHD is often linked to individual suffering and decreased quality-of-life, difficulties in relationships, performance, realistic self-evaluation, moreover, without receiving diagnosis and treatment there is an increased risk of comorbid disorders, accidents, and last but not least, it results in high social costs [32]. Therefore, adequate and accessible screening possibilities, and cooperation with treatment can be crucial. The neuropsychological profile of aADHD is characterized by high heterogeneity, and the deficits of the symptom-based subgroups are different, which underscores the importance of individual-based treatment plans. Moreover, our results highlight the need for a holistic approach when assessing weaknesses and strengths in neurocognitive performance, including subjective and objective measures. Self-reported measures are important because they reflect the patients’ experience, while objective neuropsychological measures can be related more closely to functional impairments. Furthermore, the severity of neuropsychological alterations can predict the long term outcome including overall functioning and symptom severity [20, 21]. These aspects may also facilitate treatment-adherence, including pharmacological and non-pharmacological interventions.

## Supplementary Information

Below is the link to the electronic supplementary material.

Supplementary file1 (DOCX 16 kb)

## Data Availability

The data underlying this article are available on reasonable request.
