# Supplementary material for: Differential neurocognitive profiles in adult attention-deficit/hyperactivity disorder subtypes revealed by the Cambridge Neuropsychological Test Automated Battery
Source: Eur Arch Psychiatry Clin Neurosci. 2023 Nov 18;274(7):1741–58. doi: 10.1007/s00406-023-01702-x (PMC11422285; doi:10.1007/s00406-023-01702-x)
Supplement: Supplementary file 1 — Supplementary file1 (DOCX 16 kb) [file 406_2023_1702_MOESM1_ESM.docx]

**Supplementary Table 1.** Detailed presentation of psychiatric comorbidity in the aADHD subtypes. (Chi-square test p= 0.383)

| **Comorbid diagnosis** | | **Subtypes** | | | **Total** |
| --- | --- | --- | --- | --- | --- |
|  |  | **Inattentive**  **(N=27)** | **Hyperactive-Impulsive**  **(N=5)** | **Combined**  **(N=29)** |  |
|  | No | 17 | 2 | 16 | 35 |
|  | Dylexia/dysgraphia | 1 | 0 | 3 | 4 |
|  | Depressive disorders | 6 | 1 | 7 | 14 |
|  | Anxiety disorders | 3 | 2 | 4 | 9 |
|  | Bipolar Affective Disorder | 1 | 0 | 1 | 2 |
|  | Personality disorder | 1 | 0 | 1 | 2 |
|  | Substance use | 1 | 0 | 2 | 3 |
|  | Other** | 0 | 0 | 2 | 2 |

*6 patients had two DSM-IV diagnoses based on the M.I.N.I. diagnostic interview and 2 patients had three diagnosis based on M.I.N.I. ** One subject with sleeping disorder and one person with bulimia nervosa.

**Supplementary Table 2.** Output of step-wise discriminant analyses.

| **GROUP (ADHD, HC)** | | | | | | | | |  |
| --- | --- | --- | --- | --- | --- | --- | --- | --- | --- |
| **Step** | **Entered**  **factor** | **Removed** | **Partial**  **R-square** | **F** | **p** | **Wilks Lambda** | **p (Lambda)** | **AveSqCanCor^a^** | **p**  **(ASCC^a^)** |
| 1 | RVP1 |  | 0,0899 | 11,16 | **0,0011** | 0,91 | **0,0011** | 0,0899 | **0,0011** |
| 2 | RTI1 |  | 0,0225 | 2,58 | 0,1108 | 0,89 | **0,0014** | 0,1104 | **0,0014** |
|  |  |  |  |  |  |  |  |  |  |
|  |  |  |  |  |  |  |  |  |  |
| **Subtype (ADHD-I, ADHD-MIX, HC)** | | | | | | | | |  |
| **Step** | **Entered**  **factor** | **Removed** | **Partial**  **R-square** | **F** | **p** | **Wilks Lambda** | **p (Lambda)** | **AveSqCanCor^a^** | **p**  **(ASCC^a^)** |
| 1 | RVP1 |  | 0,1002 | 6,24 | **0,0026** | 0,89 | **0,0026** | 0,0501 | **0,0026** |
| 2 | RTI1 |  | 0,0406 | 2,35 | 0,0996 | 0,86 | **0,0025** | 0,0700 | **0,0026** |
| 3 | SWM1 |  | 0,0389 | 2,22 | 0,1132 | 0,82 | **0,0020** | 0,0882 | **0,0020** |
|  |  |  |  |  |  |  |  |  |  |
| **Only ADHD (ADHD-I, ADHD-MIX)** | | | | | | | | |  |
| **Step** | **Entered**  **factor** | **Removed** | **Partial**  **R-square** | **F** | **p** | **Wilks Lambda** | **p (Lambda)** | **AveSqCanCor^a^** | **p**  **(ASCC^a^)** |
| 1 | SWM1 |  | 0,0566 | 3,36 | 0,0721 | 0,94 | 0,0721 | 0,0565 | 0,0721 |
| 2 | RTI1 |  | 0,0380 | 2,18 | 0,1459 | 0,90 | 0,0693 | 0,0924 | 0,0693 |
| 3 | IED1 |  | 0,0514 | 2,93 | 0,092 | 0,86 | **0,0427** | 0,1391 | **0,0427** |
| 4 |  | SWM1 | 0,0320 | 1,79 | 0,1864 | 0,88 | **0,0398** | 0,1106 | **0,0398** |

*^a^ ASCC= AveSqCanCor = Average Squared Canonical Correlation*
